# Experimental Evidence Shows Salubrinal, an eIF2α Dephosphorylation Inhibitor, Reduces Xenotoxicant-Induced Cellular Damage

**DOI:** 10.3390/ijms160716275

**Published:** 2015-07-17

**Authors:** Masato Matsuoka, Yuta Komoike

**Affiliations:** Department of Hygiene and Public Health I, Tokyo Women’s Medical University, Tokyo 162-8666, Japan; E-Mail: komoike@research.twmu.ac.jp

**Keywords:** apoptosis, ATF4, cytoprotection, drugs, eIF2α, environmental pollutants, ER stress, salubrinal, xenotoxicants

## Abstract

Accumulating evidence indicates that endoplasmic reticulum (ER) stress and the subsequent unfolded protein response (UPR) are involved in the pathogenesis of not only the protein misfolding disorders such as certain neurodegenerative and metabolic diseases, but also in the cytotoxicity of environmental pollutants, industrial chemicals, and drugs. Thus, the modulation of ER stress signaling pathways is an important issue for protection against cellular damage induced by xenotoxicants. The substance salubrinal has been shown to prevent dephosphorylation of the eukaryotic translation initiation factor 2 alpha (eIF2α). The phosphorylation of eIF2α appears to be cytoprotective during ER stress, because inhibition of the translation initiation activity of eIF2α reduces global protein synthesis. In addition, the expression of activating transcription factor 4 (ATF4), a transcription factor that induces the expression of UPR target genes, is up-regulated through alternative translation. This review shows that salubrinal can protect cells from the damage induced by a wide range of xenotoxicants, including environmental pollutants and drugs. The canonical and other possible mechanisms of cytoprotection by salubrinal from xenotoxicant-induced ER stress are also discussed.

## 1. Introduction

The endoplasmic reticulum (ER), an essential intracellular organelle, is responsible for the synthesis, some post-translational modification, and the delivery of biologically active proteins [1,2]. It is also critical for Ca^2+^ homeostasis. The accumulation of unfolded proteins in the lumen of this organelle causes ER stress and induces a coordinated adaptive program called the unfolded protein response (UPR) [3]. The primary functions of the UPR are to facilitate cellular adaption to environmental changes and to re-establish normal ER functions [4]. The UPR alleviates stress by inducing the expression of ER chaperones and other factors involved in ER-associated protein degradation (ERAD), and by inhibiting protein synthesis [5,6]. Under cellular stress, the ER activates three branches of the UPR: (i) the double-stranded RNA-activated protein kinase-like ER kinase (PERK)—eukaryotic translation initiation factor 2 alpha (eIF2α) pathway; (ii) the inositol-requiring enzyme 1 (IRE1)—X-box binding protein 1 (XBP1) pathway; and (iii) the activating transcription factor 6 (ATF6) pathway [7]. However, if the ER stress is prolonged and severe, the UPR can result in cell death through the activation of multiple apoptotic signaling cascades, including the CCAAT/enhancer-binding protein homologous protein (CHOP, also known as growth arrest and DNA damage gene 153 (GADD153))-mediated pathway, IRE1/tumor necrosis factor receptor-associated factor 2 (TRAF2)-mediated pathway, and Ca^2+^-dependent pathway [4].

**Figure 1:**
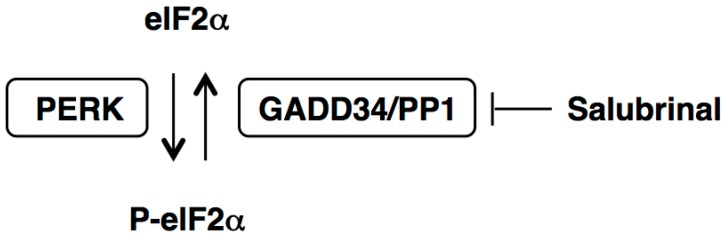
Inhibition of eIF2α dephosphorylation by salubrinal. During ER stress, the double-stranded RNA-activated protein kinase-like ER kinase (PERK) phosphorylates the eukaryotic translation initiation factor 2 alpha (eIF2α). Salubrinal prevents eIF2α dephosphorylation by inhibiting the protein complex GADD34/PP1, which consists of the general cellular serine/threonine protein phosphatase 1 (PP1) and the non-enzymatic cofactor growth arrest and DNA damage gene 34 (GADD34).

Accumulating evidence indicates that the UPR is involved in the pathogenesis of not only protein misfolding disorders, such as several neurodegenerative and metabolic diseases (Parkinson’s disease, Alzheimer’s disease, diabetes, hepatic steatosis, *etc.*) [7], but also in the cytotoxicity of environmental pollutants, industrial chemicals, and drugs [8]. Thus, the modulation of ER stress signaling pathways is an important issue for the protection against cellular damage induced by xenotoxicants. In a screen for compounds that protect PC12 rat pheochromocytoma cells from ER stress-mediated apoptosis induced by the protein glycosylation inhibitor tunicamycin, Boyce *et al.* identified a small molecule, termed salubrinal [9]. During ER stress, PERK, an ER-resident transmembrane protein, oligomerizes and phosphorylates eIF2α at serine 51 [3]. Salubrinal has been shown to prevent eIF2α dephosphorylation by inhibiting the protein complex GADD34/protein phosphatase 1 (PP1), which consists of the general cellular serine/threonine phosphatase PP1 and the non-enzymatic cofactor GADD34 [9,10] (Figure 1). The eIF2α phosphorylation appears to be cytoprotective during ER stress by inhibiting the translation initiation activity of eIF2α, which reduces global protein synthesis and results in a reduction of the ER workload [11]. Notably, activating transcription factor 4 (ATF4), a transcription factor that induces the expression of UPR target genes, is produced through alternative translation and thus not inhibited by phosphorylation of eIF2α [4,11]. Therefore, salubrinal appears to be a candidate compound for cytoprotection following exposure to the xenotoxicants that induce ER stress in their target tissues or cells.

Based on our data in a previous study [12] and the literature, we have reviewed the cytoprotective potential of salubrinal against target cell damage induced by the exposure to xenotoxicants known to induce ER stress, including environmental pollutants (cadmium, arsenic, paraquat, rotenone, benzo[a]pyrene-7,8-diol-9,10-epoxide, 2,3,7,8-tetrachlorodibenzo-*p*-dioxin, and cigarette smoke) and drugs (cisplatin and cyclosporine).

## 2. Effects of Salubrinal on Xenotoxicant-Induced Cellular Damage

### 2.1. Cadmium

Cadmium is an important occupational and environmental pollutant that damages various organs, particularly renal proximal tubular cells. We have found that cadmium chloride (CdCl_2_, 10 µM) exposure induces the expression of ATF4 and 78-kDa glucose-regulated protein (GRP78, also known as immunoglobulin heavy-chain-binding protein (BiP)), an ER-resident molecular chaperone, and phosphorylation of eIF2α in LLC-PK_1_ porcine renal epithelial cells [13]. The knockdown of GRP78 expression using short-interference RNA (siRNA) enhances CdCl_2_-induced cellular damage, indicating that the ER stress response plays a role in the protection against cadmium nephrotoxicity.

However, when LLC-PK_1_ cells are exposed to 20 µM CdCl_2_ for 4 h in the presence of 50 µM salubrinal, the percentages of round cells and Hoechst staining-positive apoptotic cells are decreased without enhancing the phosphorylation of eIF2α or affecting the expression of GRP78 and CHOP [14,15]. To explore the mechanisms underlying the cytoprotection by salubrinal, we examined apoptotic cell death and ER stress-signaling events in HK-2 human renal proximal tubular cells treated with salubrinal prior to cadmium exposure [12]. Treatment of HK-2 cells with 1–50 µM salubrinal for up to 24 h does not induce clear cellular damage, indicating low cytotoxicity of salubrinal. As expected, the levels of phosphorylated eIF2α and ATF4 proteins are elevated in HK-2 cells incubated with 20 µM salubrinal for 8 or 16 h, respectively. Using phase-contrast microscopy and the trypan blue exclusion cell viability assay, we observed that 20 µM salubrinal suppresses cellular damage and cell death following exposure to 10 or 20 µM CdCl_2_ for 24 h. Pretreatment with salubrinal reduces the number of terminal deoxynucleotidyl transferase dUTP nick end labeling (TUNEL)-positive cells and the cleavages of caspase-3 and its substrate poly(ADP-ribose) polymerase (PARP), but does not increase light chain 3B (LC3B) expression, indicating protection from cadmium-induced apoptosis but not autophagy. Although salubrinal prevents the reduction of phosphorylated eIF2α following exposure to 20 µM CdCl_2_ for 24 h, the expression of ATF4 is not increased at this time. However, the expression of ATF4 appears to be indispensable for full protection by salubrinal against cadmium cytotoxicity, because CdCl_2_-induced cellular damage is not suppressed fully in ATF4-deficient cells. Treatment with salubrinal reduces the expression of pro-apoptotic CHOP protein, but does not further enhance the expression of anti-apoptotic GRP78 protein, whereas both of these are up-regulated by ATF4 [16,17]. In addition, pretreatment of HK-2 cells exposed to 20 µM CdCl_2_ for 2–16 h with salubrinal reduces the levels of the phosphorylated form of c-Jun NH_2_-terminal kinase (JNK) and p38 but not of extracellular signal-regulated protein kinase (ERK) [12]. Because the phosphorylation of JNK and p38, which are members of mitogen-activated protein kinase (MAPK) family, stimulates apoptotic cell death [18], these findings suggest that salubrinal protects cadmium-exposed proximal tubular cells from apoptosis by suppressing cell death signal transduction pathways. Additional investigations will be needed to clarify whether salubrinal can directly or indirectly act on the IRE1/TRAF2/apoptosis signal-regulating kinase 1 (ASK1) cascade that leads to the activation of JNK and p38 [19].

### 2.2. Arsenic

Intoxication with arsenic causes peripheral neuropathy, vascular diseases, and skin cancer in humans. Effects of salubrinal treatment on arsenic-induced cytotoxicity have been examined in rat dorsal root ganglion (DRG) explants [20] and SVEC4-10 mouse endothelial cells [21]. Co-incubation with 30 µM salubrinal for 24 h significantly attenuates sodium arsenite (NaAsO_2_, 30 µM)-induced reduction in procaspase-12 levels and DNA fragmentation in rat DRG explants [20]. In rodents, caspase-12 is associated with the ER membrane and is activated by the IRE1/TRAF2 pathway [4]. These findings suggest that salubrinal suppresses arsenic-induced neuronal ER stress and subsequent apoptosis through the inactivation of caspase-12. It remains to be determined whether the protective effects of salubrinal against arsenic neurotoxicity depend on the inhibition of eIF2α dephosphorylation.

In SVEC4-10 cells, pretreatment with 10 µM salubrinal for 16 h slightly enhances GRP78 and CHOP expression following exposure to 7.5 µM arsenic trioxide (As_2_O_3_) for an additional 16 h [21]. Conversely, salubrinal (10 and 20 µM) increases the MTT assay-based cell viability of SVEC4-10 cells after 24 h incubation with 7.5 µM As_2_O_3_. The effects of salubrinal treatment on the levels of phosphorylated eIF2α and ATF4 were not examined in this study. While the contribution of downstream molecules of ATF4 is not clear, these results show that salubrinal protects endothelial cells as well as DRG explants from arsenic-induced cellular damage.

### 2.3. Paraquat

Acute intoxication with paraquat (*N*,*N*ʹ-dimethyl-4,4ʹ-bipyridinium dichloride), a widely used herbicide, causes severe lung injury in humans, resulting in death. The therapeutic effects of salubrinal administration were investigated in the rat acute paraquat poisoning model [22]. Injection of salubrinal intraperitoneally (i.p.) on days 1, 3, and 5 at a dose of 0.5 mg/kg/day inhibits hemorrhage and fibrosis, but promotes inflammatory infiltration in the lung tissues of rats given paraquat (40 mg/kg, intra-gastral). Furthermore, salubrinal reduces LC3 and increases Bcl-2, an apoptosis inhibitor, in this animal model. The mechanism of protection by salubrinal from paraquat-induced pulmonary damage, apoptosis, and autophagy needs further exploration for its application as an antidote to exposure.

Paraquat is also known to be a dopaminergic neurotoxin and has been used to investigate the pathogenesis of Parkinson’s disease. Pretreatment of SH-SY5Y human neuroblastoma cells with 10 µM salubrinal for 24 h significantly attenuates cellular damage induced by subsequent exposure to 0.5 mM paraquat for 48 h [23]. Paraquat-induced expression of ER stress biomarkers, including GRP78, ER degradation-enhancing α-mannosidase-like protein (EDEM), and CHOP, are inhibited effectively by salubrinal. Furthermore, the phosphorylation of IRE1, ASK1, JNK, and the increase in caspase-3 activity are inhibited by salubrinal. These findings show that salubrinal may inhibit the IRE1/TRAF2/ASK1/JNK cascade associated with apoptosis in SH-SY5Y cells exposed to paraquat. In another study using SH-SY5Y cells, pretreatment with 20 µM salubrinal for 1 h rescues cells from apoptotic events (release of cytochrome c from mitochondria into the cytosol, caspase-3 activation, and nuclear fragmentation) induced by exposure to 0.1 mM paraquat for an additional 24 h [24]. However, salubrinal treatment exacerbates autophagy in ASK1-overexpressing cells in both the presence and absence of paraquat, which may be a protective response. These two studies suggest that salubrinal can protect dopaminergic cells from paraquat cytotoxicity by affecting the signaling pathways leading to apoptosis and/or autophagy.

### 2.4. Rotenone

Rotenone, a pesticide, causes neurotoxicity through mitochondrial complex I inhibition and has been used to induce an experimental model of Parkinson’s disease. Co-incubation with 0.1 µM rotenone and 40 µM salubrinal for 12 h attenuates rotenone-induced caspase-3 activation, apoptotic cell death, and the reduction of cell viability in SH-SY5Y cells [25]. The presence of salubrinal enhances rotenone-induced eIF2α phosphorylation and downstream ATF4 expression. Although salubrinal does not decrease CHOP level in rotenone-treated SH-SY5Y cells, the level of C/EBPβ isoform liver inhibitory protein (LIP), which is required for nuclear translocation of CHOP during ER stress, is reduced by salubrinal treatment, resulting in the reduction of CHOP level in nuclear extracts.

The transfection of ATF4 siRNA into SH-SY5Y cells diminishes the protective effect of salubrinal against rotenone-induced cell death. In addition, the level of parkin protein, which is up-regulated by ATF4 and protects cells from ER stress-induced neuronal cell death, decreases after treatment with rotenone, whereas this reduction is counteracted by treatment with salubrinal. These findings suggest that the ATF4/parkin pathway is responsible for salubrinal-mediated protection from dopaminergic cell death induced by rotenone.

In another neuroblastoma cell line, mouse neuro-2A cells, the protective effects of salubrinal against rotenone-induced ER stress also have been demonstrated [26]. Pretreatment of neuro-2A cells with 25 µM salubrinal for 30 min inhibits eIF2α dephosphorylation following the exposure to 0.5 or 1 µM rotenone for 16 h. The rotenone-induced cellular damage, reactive oxygen species (ROS) generation, GRP78 and CHOP expression, DNA damage, and caspases-12 and -3 activation are suppressed by salubrinal treatment. However, no protection is observed for the decreased mitochondrial membrane potential and caspase-9 activation that are induced by rotenone. These findings suggest that salubrinal reduces rotenone-induced ER stress but might not affect the mitochondrial dysfunction in neuroblastoma cells.

### 2.5. Benzo[a]pyrene-7,8-diol-9,10-epoxide

Benzo[a]pyrene-7,8-diol-9,10-epoxide (BPDE) is the active metabolite of benzo[a]pyrene, a carcinogenic polycyclic aromatic hydrocarbon present in the environment. The BPDE-induced DNA damage blocks cell cycle progression and induces apoptosis. Pretreatment of human amniotic epithelial cells with 20 µM salubrinal for 30 min prolongs BPDE (0.5 and 1 µM)-induced eIF2α phosphorylation until 48 h after exposure [27]. Salubrinal attenuates cell cycle arrest after 24 or 30 h, and DNA fragmentation and nuclear condensation after 48 h exposure to 0.5 µM BPDE. In addition, salubrinal partially suppresses BPDE-induced reduction of cell viability after 24 or 48 h exposure. These results indicate that salubrinal can maintain eIF2α phosphorylation, attenuate apoptosis, and promote cell survival in amniotic epithelial cells exposed to BPDE.

### 2.6. 2,3,7,8-Tetrachlorodibenzo-p-dioxin

The most toxic dioxin, 2,3,7,8-tetrachlorodibenzo-*p*-dioxin (TCDD), is known to have significant neurotoxicity. Pretreatment of differentiated PC12 rat pheochromocytoma cells with 1, 5, and 30 µM salubrinal for 1 h inhibits the decrease in cell viability induced by exposure to 200 nM TCDD for 24 h in a dose-dependent manner [28]. Furthermore, co-treatment of PC12 cells with 30 µM salubrinal and 200 nM TCDD for 24 h decreases the number of apoptotic cells. This anti-apoptotic effect is accompanied by an increase in eIF2α phosphorylation and GRP78 expression, and a decrease in CHOP and active caspase-3 expression. In contrast, TCDD exposure induces the phosphorylation of PERK, and siRNA knockdown of eIF2α protein enhances TCDD-induced cell death. Thus, salubrinal may protect against TCDD-induced neuronal apoptosis through the PERK/eIF2α pathway, whereas the pathophysiological roles of its downstream molecules, ATF4, GRP78, and CHOP, are yet to be defined.

### 2.7. Cigarette Smoke

Cigarette smoke is well known to play a major role in the pathogenesis of chronic obstructive pulmonary disease (COPD). Prior to the treatment of normal human bronchial epithelial (HBE) cells with 5% cigarette smoke extract (CSE) for 24 h, incubation with 1–100 µM salubrinal for 2 h suppresses CSE-induced apoptosis in a dose-dependent manner accompanied by a decrease in caspases-3 and -4 expression [29]. While CSE-induced phosphorylation of PERK and eIF2α peaks at 6 h and returns to the normal levels at 24 h, salubrinal maintains phosphorylated eIF2α levels without affecting the phosphorylated PERK level after 24 h exposure to CSE. In addition, experiments using PERK siRNA indicate that salubrinal protects HBE cells against CSE insults through maintaining the homeostasis of the PERK/eIF2α pathway.

The expression of valosin-containing protein, which plays a role in both protein extraction from the ER and the ubiquitin-proteasome mediated protein degradation by ERAD, is suppressed in the lungs of mice treated with salubrinal (1 mg/kg, intra-tracheally) for the last 24 h of the 3 days cigarette smoking protocol [30]. It will be interesting to investigate pathophysiologically whether salubrinal is a candidate substance for the effective treatment of COPD.

The cytoprotective effects of salubrinal have also been reported in human periodontal ligament cells exposed to nicotine [31], the *in vitro* model of periodontitis. Pretreatment with 10 µM salubrinal for 1 h suppresses nicotine-induced necrotic cell death, the expression of GRP78, CHOP, matrix metalloproteinases (MMPs-1, -2, -8, and -9), and the down-regulation of extracellular matrix molecules (type I collagen, elastin, and fibronectin) in periodontal ligament cells incubated with 10 mM nicotine for 24 h. In addition, nicotine-induced activation of Akt, MAPKs (ERK, JNK, and p38), and nuclear factor-kappa B (NF-κB) is suppressed by salubrinal treatment followed by the incubation with 10 mM nicotine for 30 min. Further studies are needed to reveal the role of these multiple signal transduction pathways in nicotine-induced cell death and its attenuation by salubrinal treatment.

### 2.8. Cisplatin

Cisplatin, the widely used anti-cancer drug, has many adverse side effects, the most important of which is nephrotoxicity (*i.e.*, renal tubular cell injury and death). Pretreatment of NRK52E rat renal proximal tubular cells with 50 µM salubrinal for 1 h suppresses apoptotic cell death induced by the exposure to 50 µM cisplatin for 24 h [32]. Cisplatin-induced apoptosis is enhanced by the transfection of GRP78 siRNA into NRK52E cells, suggesting that ER stress-mediated up-regulation of GRP78 expression acts as a survival mechanism for the rescue of cisplatin-induced renal tubular cell damage.

Unexpectedly, the *in vivo* experiments conducted by the same investigators show that salubrinal administration aggravates cisplatin-induced renal cell injury in the mouse model [33]. Salubrinal (1 mg/kg/day, i.p.) administered 1 h before treatment with cisplatin (10 mg/kg/day, i.p.) for 2 consecutive days enhances cisplatin-induced blood biochemical and renal histomorphological alterations. In addition, salubrinal significantly enhances eIF2α phosphorylation, the up-regulation of ATF4, CHOP, and pro-apoptotic Bax proteins, the down-regulation of anti-apoptotic Bcl-2 protein, cleavage of caspases-12, -9 and -3, and oxidative stress in the kidneys of cisplatin-treated mice. Treatment with *N*-acetylcysteine, the ROS scavenger, could reverse the cisplatin nephrotoxicity enhanced by salubrinal, suggesting the possible involvement of oxidative stress in these deleterious effects from salubrinal.

### 2.9. Cyclosporine

Cyclosporine, the calcineurin inhibitor, is an effective immunosuppressant, which, however, has nephrotoxic potential. Treatment with 50 µM salubrinal protects normal human renal epithelial cells against cyclosporine (6 µM)-induced epithelial phenotypic changes and apoptotic cell death after 24–72 h exposure [34]. Salubrinal (50 µM) significantly reduces the expression of GRP78 and GADD34, and also blocks LC3II formation in cyclosporine (8 µM)-treated tubular cells [35]. In the rat model of cyclosporine (15 mg/kg/day, subcutaneously, 28 days)-induced nephrotoxicity, treatment with salubrinal (1 mg/kg/day, i.p., 28 days) also improves renal dysfunction and morphological changes and reduces the expression of GRP78 and calreticulin, another ER stress marker, in the rat kidneys [34]. These *in vitro* and *in vivo* experiments demonstrate that salubrinal could repress the nephrotoxicity of cyclosporine by relieving ER stress.

In addition to tubular epithelium, the protective effects of salubrinal against cyclosporine-induced insults were also shown in human umbilical artery endothelial cells (HUAECs) [36]. In comparison to HUAECs treated with 10 µM cyclosporine alone for 24–72 h, co-incubation with 25 µM salubrinal reduces GRP78 expression and confers protection from cyclosporine-induced endothelial cell death and phenotypic changes.

## 3. Possible Mechanisms of Protection by Salubrinal from Xenotoxicant-Induced Cellular Damage

**Figure 2:**
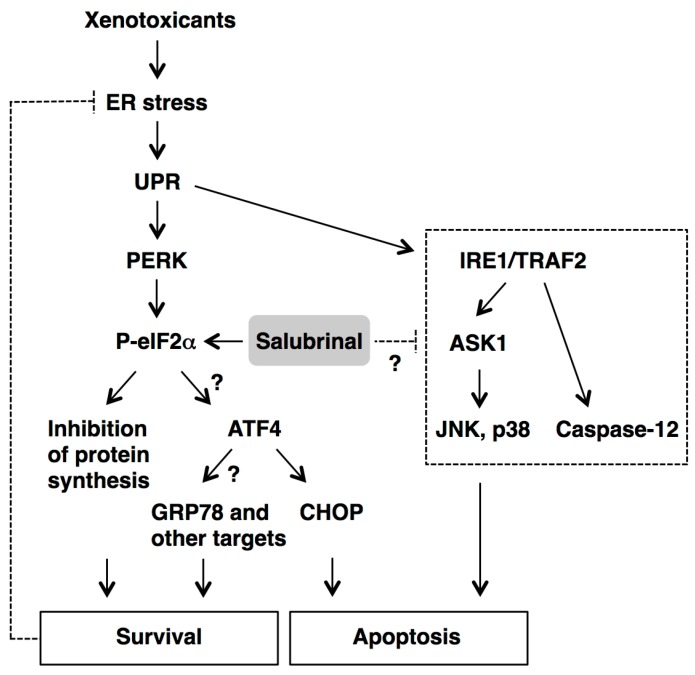
Proposed effects of salubrinal on the xenotoxicant-activated ER stress signaling pathways. Following exposure to xenotoxicants, ER stress activates the double-stranded RNA-activated protein kinase-like ER kinase (PERK)/eukaryotic translation initiation factor 2 alpha (eIF2α) pathway and the inositol-requiring enzyme 1 (IRE1)/tumor necrosis factor receptor-associated factor 2 (TRAF2) pathway, which are the two branches of the unfolded protein response (UPR). The enhancement or prolongation of eIF2α phosphorylation by salubrinal reduces global protein synthesis and the overall amount of ER stress, resulting in cell survival. In contrast to most proteins, the expression of activating transcription factor 4 (ATF4) is up-regulated through alternative translation and thus not impacted by the reduction in protein synthesis. The ATF4 protein balances between the signals leading to survival (by 78-kDa glucose-regulated protein (GRP78) and other UPR targets) and to apoptosis (by CCAAT/enhancer-binding protein homologous protein (CHOP)). Salubrinal might act on the IRE1/TRAF2 pathway that leads to the activation of apoptosis signal-regulating kinase 1 (ASK1)/c-Jun NH_2_-terminal kinase (JNK)-mediated, ASK1/p38-mediated, and caspase-12-dependent apoptotic pathways.

The experiments, mainly performed *in vitro*, show that pretreatment or co-incubation with 1–100 µM salubrinal can suppress some xenotoxicant-induced target cell damage and apoptosis. However, the mechanisms underlying the cytoprotection conferred by salubrinal are extremely complex, and might differ depending on the nature and the severity of the insult, as well as the cell type being investigated. The enhancement or prolongation of xenotoxicant-induced eIF2α phosphorylation is observed in a number of experiments [12,25,26,27,28,29]; this concurs with the ability of salubrinal to prevent eIF2α dephosphorylation [9]. The resultant inhibition of global protein synthesis alleviates the workload on the ER, and subsequently reduces energy consumption, leading to cell survival (Figure 2). This beneficial effect of salubrinal might be reflected by a resultant decrease in the levels of ER stress markers, including GRP78 [23,26,31,34,35,36], calreticulin [34], GADD34 [35], and CHOP proteins [12,23,26,28,31].

In contrast to most proteins, the expression of ATF4 is up-regulated because it has upstream open reading frames in its 5ʹ untranslated region, which are bypassed only when eIF2α is phosphorylated at serine 51 [6,37]. Although the elevation of ATF4 level by salubrinal treatment is clearly demonstrated in only one study [25], the experiments using ATF4 siRNA show that ATF4 expression is a prerequisite for the full cytoprotection conferred by salubrinal [12,25]. Because ATF4 could balance between the signals leading to survival (by GRP78 and other UPR targets) and to apoptosis (by CHOP), the magnitude or duration of eIF2α phosphorylation appears to be the key factor for determining the net effects of salubrinal on cell fate (Figure 2). The GRP78 protein is an ER-resident molecular chaperone that prevents the aggregation of unfolded or misfolded proteins, so that they can be properly re-folded [1,3,38]. Although the knockdown of GRP78 exacerbates the cellular damage induced by cadmium [13] and cisplatin [32], it is inconclusive whether salubrinal exerts the cytoprotective effects through the up-regulation of GRP78 expression [21,28]. Further studies are required to reveal other downstream target molecules activated by ATF4.

Another possible mechanism of salubrinal is to suppress the xenotoxicant-activated signaling pathways leading to cell death. The recruitment of TRAF2 by phosphorylated IRE1, one of the branches of the UPR, activates both the ASK1/JNK-mediated and caspase-12-dependent apoptotic pathways [2,4] (Figure 2). Salubrinal inhibits JNK phosphorylation induced by cadmium [12], paraquat [23], and nicotine [31], as well as caspase-12 activation induced by arsenic [20] and rotenone [26]. The effects of salubrinal on the IRE1/TRAF2 pathway might be indirect, and could involve reducing the overall amount of ER stress through the inhibition of eIF2α dephosphorylation (*i.e.*, activation of PERK/eIF2α pathway). On the other hand, activation of PERK is shown to negatively regulate the IRE1/XBP1 pathway through NF-κB and microRNA-30c-2* [39]. Therefore, the regulatory crosstalk within the UPR branches should also be considered for the mechanism of salubrinal.

Conversely, mechanisms independent of eIF2α phosphorylation have been suggested for the pharmacological actions of salubrinal. Salubrinal attenuates β-amyloid-induced neuronal death through the inhibition of IκB kinase (IKK) complex phosphorylation and the subsequent NF-κB activation without affecting eIF2α phosphorylation [40]. Similarly, salubrinal reduces the levels of phosphorylated IKK and NF-κB p65, as well as the level of phosphorylated p38, without changing the level of phosphorylated eIF2α in chondrocytes treated with cytokines (tumor necrosis factor α and interleukin 1β) [41]. These findings suggest that NF-κB and p38 signaling pathways might be other targets of salubrinal. Although salubrinal could suppress the nicotine-induced activation of both NF-κB and p38 in periodontal ligament cells [31], it remains to be clarified whether salubrinal acts on these signaling pathways, which are prone to be activated by xenotoxicants. Another mechanism of cytoprotection conferred by salubrinal is the protection of the anti-apoptotic Bcl-2 protein associated with the ER. Salubrinal protects Bcl-2 protein from inactivation caused by an interaction with the non-peptidic antagonist HA14-1 and from the porphycene-induced photodamage in murine leukemia L1210 cells [42]. Because xenotoxicants can alter the expression of Bcl-2, it would be interesting to clarify whether salubrinal inhibits the apoptotic and autophagic effects by the preservation of Bcl-2 function.

## 4. Conclusions

Literature shows that salubrinal can protect cells or animals from target cell damage induced by a wide range of xenotoxicants, including environmental pollutants and drugs. Furthermore, salubrinal shows neuroprotective effects *in vitro* and *in vivo* against neurotoxic substances considered to be responsible for neurological disorders, such as β-amyloid [40,43,44,45], α-synuclein [46,47], mutant huntingtin protein [48], superoxide dismutase 1 mutant [49], ceramide [50], and kainic acid [51,52]. While the primary mechanism underlying the cytoprotection by salubrinal appears to be the inhibition of eIF2α dephosphorylation, there are still other pharmacological actions yet to be defined. In conclusion, the eIF2α dephosphorylation inhibitor salubrinal is a useful substance for the investigation of the mechanisms of ER stress-related pathogenesis, including the cellular damage induced by xenotoxicants. More extensive efforts, especially using animal models, are required to extrapolate these potential benefits of salubrinal for the protection against cellular damage induced by xenotoxicants.
